# Analysis on risk factors for neck shortening after internal fixation for Pauwels II femoral neck fracture in young patients

**DOI:** 10.1186/s40001-021-00531-9

**Published:** 2021-06-24

**Authors:** Fulong Zhao, Lijuan Guo, Xuefei Wang, Yakui Zhang

**Affiliations:** 1 Trauma Orthopedics, Beijing Luhe Hospital Affiliated To Capital Medical University, No. 82, Xinhua South road, Tongzhou District, Beijing, 101149 China; 2 Clinical Laboratory, Emergency General Hospital, Beijing, 100028 China

**Keywords:** Young patients, Femoral neck fracture, Internal fixation, Neck shortening, Risk factors

## Abstract

**Background:**

Femoral neck shortening can occur in young patients receiving internal fixation for Pauwels type II femoral neck fracture. The risk factors for neck shortening, which can affect hip function, are not clear. This study aimed to retrospectively identify risk factors for neck shortening after internal fixation with parallel partially threaded cannulated cancellous screws (FPTCS) for Pauwels type II femoral neck fracture in relatively young patients.

**Methods:**

Clinical data from 122 cases with Pauwels type II femoral neck fracture from February 2014 to February 2019 were reviewed and analyzed, and causes of neck shortening were statistically analyzed. And the Chi-squared test or Fisher’s exact test was used to compare indicators. Multivariate analysis was conducted with non-conditional logistic regression analysis.

**Results:**

Statistically significant differences were found in age, sex, BMD, BMI, fracture type, posterior medial cortex comminution, and reduction quality between patients with femoral neck shortening and those without femoral neck shortening. Logistic regression analysis showed that fracture type, posterior medial cortex comminution, and reduction quality were the main risk factors for neck shortening.

**Conclusion:**

Fracture type, posterior medial cortex comminution, and reduction quality can be used as important reference indexes to predict the possibility of neck shortening after internal fixation with FPTCS for Pauwels type II femoral neck fracture in young patients. BMD and BMI may be also risk factors.

## Background

Femoral neck fracture is a common injury in orthopedics. Young patients account for 2–3% of cases [1], and 6000 young people have femoral neck fracture every year [2]. The fracture of the femoral neck in young patients is often caused by high-energy trauma, the increasing incidence of which has increased the incidence of femoral neck fracture in young patients. The treatment is anatomical reduction and internal fixation as soon as possible [3, 4]. The failure rate of treatment of femoral neck fracture in young adults is high. This is a challenging problem because there are many treatment options and consensus on the best treatment has been difficult to reach [5]. Multiple cancellous screws or sliding hip screws are still the most commonly used internal fixation implants [6, 7]. Young patients are commonly treated by fracture fixation with FPTCS [8–11], while fully threaded screws are rarely used [12].

Pauwels type II fracture is described as an unstable fracture: shear stress at the fracture end may have an adverse effect on fracture healing, but internal fixation can reduce shear stress and promote fracture healing [13]. However, in treatment of femoral neck fracture in the young, shortening of the femoral neck can occur after fixation with multiple cancellous screws, and this affects the function of the hip joint [14, 15]. This has led to more research on the incidence and resolution of this complication [16, 17]. Other studies have focused on the complications of this operation [18–21]. So far, few studies have attempted to determine the risk factors for shortening by using multivariate regression analysis.

In this retrospective study, fixations for Pauwels type II fracture were performed based on the inverted triangle configuration. An inverted triangle screw configuration has been recommended for internal fixation in femoral neck fractures, because this configuration has provided better biomechanical stability compared to other configurations [22–24]. Based on femur anatomy, the cross section of the femur neck is similar to an inverted triangle configuration. Therefore, our screw configuration was demonstrated to provide clear mechanical advantages in previous studies [25, 26].However, some studies indicated that different operative protocols for femoral neck fractures had different effects on the mechanical features [27–29].

Fully understanding the risk factors for neck shortening after internal FPTCS for young Pauwels type II femoral neck fracture is of great importance in improving the safety of the operation and ensuring its therapeutic effect. In this study, we retrospectively analyzed the main causes of neck shortening after internal FPTCS in young patients with Pauwels type II femoral neck fracture in order to identify risk factors, and tried to determine the effects of these factors on postoperative neck shortening.

## Patients and samples

### Criteria for inclusion and exclusion of patients

Criteria for patient inclusion in the study were age 59 years or younger, Pauwels type II femoral neck fracture confirmed by X-ray and CT, capacity for basic self-care, normal cognition, and the patient’s agreement to participate in the study. Exclusion criteria were pathological fracture, multiple fracture, and mental illness.

### Research objectives

The clinical data from 122 young patients with femoral neck fractures treated in our hospital from February 2014 to February 2019 were retrospectively analyzed. The study included 65 males (53.28%) and 57 females (46.72%), and the average age was 40.79 ± 19.63 years (range 18–59 years). According to the Garden classification, there were 14 cases with type I, 25 with type II, 45 with type III, and 38 of type IV. Conditions before the patient’s injury were fully understood. Relevant examinations were performed in preoperative assessment. All operations were performed by the same group of surgeons using three FPTCS. Reduction quality was assessed using the method of Lowell [30].

### Surgical methods

**Fig. 1:**
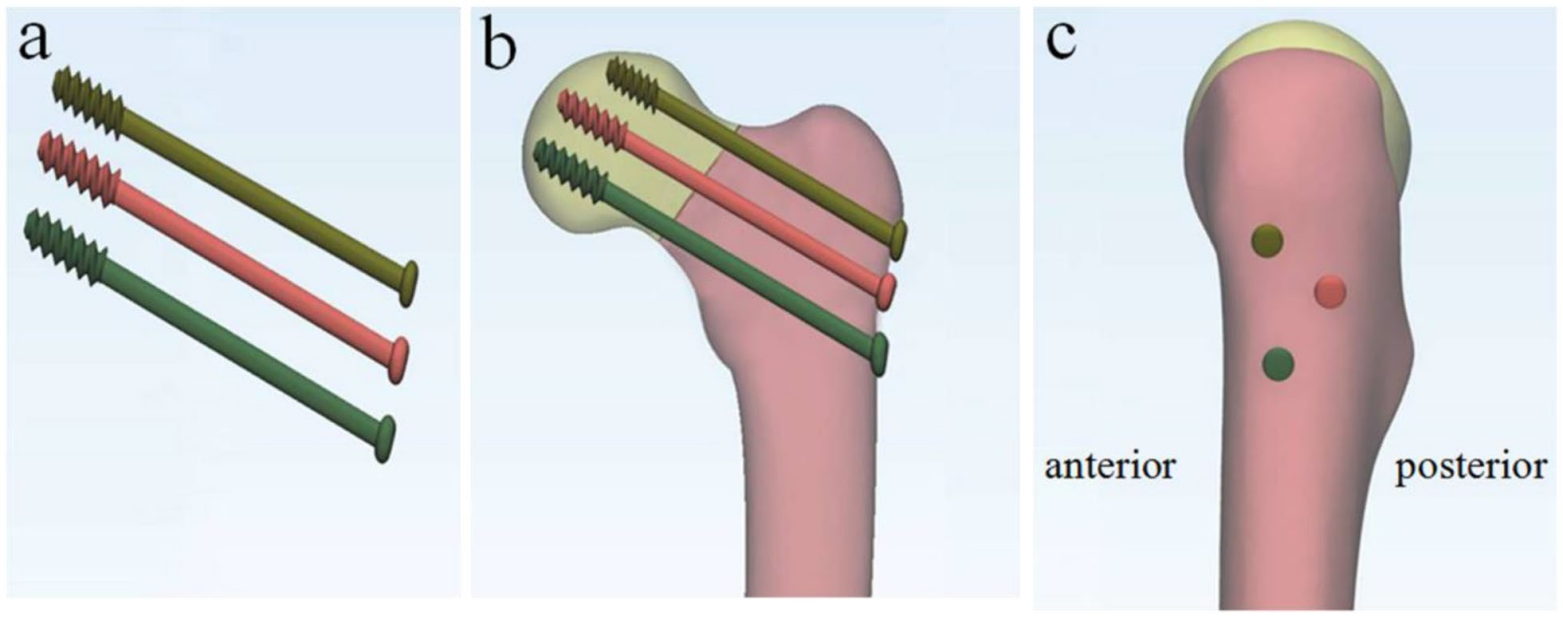
**a** Three parallel partially threaded cannulated cancellous screws. **b**, **c** Anteroposterior and lateral schematic diagram

The patient was placed on a traction bed and their limbs were fixed. C-arm X-ray was used to identify whether fracture reduction was satisfactory or not in the anteroposterior (A/P) and lateral views. Based on satisfactory fracture reduction, three FPTCS (7.0 mm, titanium alloy, American General Corporation, USA), were implanted in the femoral neck in an inverted triangle configuration: the inferior screw was placed on the coronal axis of the proximal femur, close to the femoral calcar; the superior screw was placed in the anterior superior cortex of the femoral neck; and the third screw was placed in the posterior cortex for support (Fig. 1). All three screws were placed under the subchondral bone of the femoral head. The quality of fracture reduction was judged by postoperative examination of the hip joint in the A/P and lateral views.

### Research methods

The method used to measure neck shortening in this study is consistent with previous studies [17, 31]. According to whether it occurred, the 122 young patients with femoral neck fractures were divided into a neck shortening group (37 cases) and a non-neck shortening group (85 cases). In this study, we found that, in the neck shortening group, the femoral neck was shortened by 5–9 mm, with an average of 7 mm. Previous studies have shown that, when the femoral neck is shortened by more than 5 mm, it had a negative effect on the quality of life [14, 20, 32]. Observed indexes in the study included patient’s age, sex, BMD, body mass index (BMI) [33], fracture type, posterior medial cortex comminution, reduction quality, surgical time, time to weight-bearing, and time of hospitalization, which were entered into the database. The average value of each index was recorded.

### Statistical methods

All data analyses were conducted using SPSS 22.0 statistical software. The occurrence of neck shortening was considered as a dependent variable, while the observed indexes were used as independent variables. Data are reported as the mean ± standard deviation (SD), and were compared using the Chi-squared test or Fisher’s exact test. Non-conditional logistic regression was performed to identify risk factors for neck shortening. The difference in the effective rate was considered statistically significant at *p* < 0.05.

## Results

### Neck fracture healing rate and Harris score in the neck shortening group and non-shortening group

There were 37 (30.33%) cases in the shortening group and 85 cases in the non-shortening group. In the shortening group, 33 cases (89.19%) healed, and the average Harris score was 79 ± 13. In the non-shortening group, 74 cases (87.06%) healed, and the mean Harris score was 87 ± 16. The differences in the fracture healing rate and the mean Harris score were not statistically significant (*p* > 0.05 for each). The total incidence of nonunion was 12.29% (15/122):10.81% (4/37) in the shortening group, and 12.94% (11/85)in the non-shortening group (Table 1).

**Table 1:** Comparison of healing rate between two groups

	Shortening	Non-shortening	*χ*^2^	*p*
Healing rate	89.19% (33/37)	87.06% (74/85)	0.026	0.871
Harris score	79 ± 13	87 ± 16	0.386	0.534

### Factors influencing the occurrence of neck shortening

There were statistically significant differences in variables between the femoral neck shortening group and non-shortening group, including patient age, sex, BMD, BMI, fracture type, posterior medial cortex comminution, and reduction quality. There were no statistically significant differences between the two groups with respect to surgical time, time to weight-bearing, or hospitalization time (Table 2).

**Table 2:** Comparison of patient characteristics between the two groups

Factors	*n*	Shortening group	Non-shortening group	*χ*^2^	*p*
Age (years)					
18–44	65	14	51	5.086	0.024
45–59	57	23	34		
Sex					
Male	83	30	53	4.157	0.041
Female	39	7	32		
BMD					
T > − 2.5	75	17	58	5.408	0.020
T ≤ − 2.5	47	20	27		
BMI (kg/m^2^)					
< 24.0	60	12	48	8.745	0.012
24.0 ≤ BMI < 28.0	44	15	29		
≥ 28.0	18	10	8		
Fracture type					
Type I, II	33	19	14	15.895	0.000
Type III, IV	89	18	71		
Posterior medial cortex comminution					
Yes	82	32	50	8.952	0.002
No	40	5	35		
Reduction quality					
Grade I, II	77	15	62	11.625	0.000
Grade III, IV	45	22	23		
Surgical time (h)					
≤ 24	79	23	56	0.156	0.692
> 24	43	14	29		
Time to weight-bearing (months)					
≤ 2	91	27	64	0.073	0.787
> 2	31	10	21		
Hospitalization time (week)					
≤ 1	75	22	53	0.091	0.762
> 1	47	15	32		

### Non-conditional logistic regression analysis of factors influencing neck shortening

Between-group differences in the above seven factors were statistically significant (*p* < 0.05) by the Chi-squared test. These factors were further analyzed using logistic regression. The results showed that BMD (T ≤ –2.5), BMI (≥ 28.0 kg/m^2^), fracture type (type III, IV), posterior medial cortex comminution (Yes), and reduction quality (Grade III, IV) had an impact on the occurrence of neck shortening (Table 3).

**Table 3:** Non-conditional logistic regression analysis of factors influencing femoral neck shortening after fixation with partially threaded cannulated screws in young patients

Influencing factors	B	Exp (B)	95*%* *CI*	*p*
Age (45–59 years)	1.141	0.686	(0.85–13.59)	0.104
Sex (female)	0.761	0.653	(0.57–10.44)	0.282
BMD (T ≤ − 2.5)	3.489	10.21	(3.89–16.95)	0.007
BMI (≥ 28.0 kg/m^2^)	2.767	9.16	(2.42–15.51)	0.004
Fracture type (Garden III, IV)	3.103	10.29	(5.94–21.07)	0.000
Posterior medial cortex comminuted (yes)	5.614	27.88	(6.26–41.17)	0.000
Reduction quality (Grade III, IV)	2.056	14.94	(5.31–28.85)	0.000

*CI* confidence interval

## Discussion

Femoral neck fracture is a common fracture. Previous studies have shown that good closed reduction and internal fixation using cannulated screws in the treatment of femoral neck fracture is efficacious [34, 35]. However, neck shortening in the course of treatment is still worthy of attention. A considerable proportion of patients present this phenomenon on follow-up imaging, which is less concerned. Its incidence in this study was 30.33% (37/122), very similar to that in a previous study [17]. The total incidence of nonunion was 12.29%, in accord with previous studies [36, 37].

Here, we retrospectively studied data from 122 young patients who had Pauwels type II femoral neck fractures treated using FPTCS and analyzed possible factors influencing neck shortening. Univariate analysis showed that patient age, sex, BMD, BMI, fracture type, and reduction quality were risk factors for neck shortening. Age and BMD were closely related to the occurrence of neck shortening. With increasing age, BMD may decline, and it is clear that bone quality may determine the probability of femoral neck fracture [38]. Reduction of the axial anti-compression strength of the femoral neck leads to its shortening. Sex is also an important influencing factor. Compared with men, women have thinner bone cortex and lower bone density. After menopause, estrogen decreases rapidly, which further affects the process of fracture repair [39]. BMI also increases the risk of neck shortening after fracture [40]. Increased BMI may directly increase the axial pressure on the fracture end, leading to neck shortening. Therefore, for patients with these risk factors, a comprehensive preoperative evaluation is essential, although this complication may result from a combination of these factors.

Non-conditional logistic regression analysis showed that fracture type (Garden III, IV) and reduction quality (Grade III, IV) were the main causes of postoperative neck shortening. Higher fracture type, posterior medial cortex comminution, and lower reduction quality could indicate a greater risk of postoperative neck shortening in young patients who had Pauwels type II femoral neck fractures. Therefore, more attention should be paid to these three indicators during clinical observations. We found that fracture type (Garden III, IV) may increase the likelihood of neck shortening, as previously reported [9, 18]. Garden type III and IV fractures are unstable and often accompanied by comminution or posterior medial bone cortical defects [41]. Comminuted fractures increase bone absorption after surgery and are more likely to produce neck shortening after healing. Destruction of the posterior medial cortex often leads to a lowering of both the quality of reduction and resistance to axial loading, and results in neck shortening, even when complete anatomical reduction is achieved in the operation [42]. Garden's Alignment Index is commonly used to evaluate reduction quality. When the evaluated fracture reduction quality fails to meet the standard of anatomical reduction, the probability of postoperative neck shortening is greatly increased [18]. It is possible that the stress on the fracture’s broken end is not uniform, leading to collapse at the fracture site, with consequent neck shortening.

In this study, a univariate analysis showed that patient age, sex, BMD, BMI, fracture type, and reduction quality were risk factors for femoral neck shortening. Importantly, a multifactor analysis showed that, in the shortening group, the main causes of neck shortening were the fracture type (Garden III, IV), reduction quality (Grade III, IV), and the posterior medial cortex comminution; additionally, secondary causes might be BMD (T ≤  − 2.5) and BMI (≥ 28.0 kg/m^2^). These results indicated that the Harris score in the shortened group was lower than that in the non-shortened group. That finding suggested that neck shortening would result in reduced hip function, which was mainly manifested as claudication and poor abduction strength [43]. A potential mechanism for these manifestations could be that neck shortening might have affected the arm of the abductor muscle, which reduced the ability of the hip joint to maintain a stable gait and pelvic balance [44]. Consequently, patients had to increase the abductor muscle strength to compensate when walking, which resulted in walking claudication. In future, it would be interesting to explore whether there might be some quantitative relationship between the degree of femoral neck shortening and the hip function score.

Although early mobilization following young femoral neck fracture surgery is of superior importance, to date, there is no unified postoperative rehabilitation protocol. In this study, the postoperative weight-bearing program was determined by the physician in charge, and it depended on many factors, including age, sex, BMD, BMI, fracture type, posterior medial cortex comminution, and reduction quality. In general, partial weight-bearing activities, with the use of an assistive device, were allowed as soon as patients could tolerate them. In patients with the risk factors mentioned above, partial weight-bearing was delayed until 8 weeks after surgery. During those 8 weeks, the patient performed functional exercises for the affected limb, without bearing weight.

This study has some limitations. First, our results were based on a small number of patients. We still did not accurately predict whether neck shortening was related to the healing rate of the femoral neck fracture. Second, we did not include patients with Pauwels type I and III fractures. Third, because there are few studies on neck shortening and all methods for measuring neck shortening are still in their initial stages, more accurate methods of measurement need to be further explored.

To reduce the effect of postoperative neck shortening on hip function, we have changed our treatment of femoral neck fractures by using fully threaded screws or a femoral neck system (FNS), instead of FPTCS. Currently, a clinical follow-up is ongoing to evaluate the degree of postoperative neck shortening and its effects on hip function, when treated with fully threaded screws or FNS. Those findings will be reported in future studies.

In conclusion, Our results support the use of fracture type, the presence of comminution of the posterior medial cortex, and reduction quality as important reference indexes to predict the possibility of neck shortening after internal fixation with FPTCS for Pauwels type II femoral neck fracture in young patients. BMD and BMI may also be risk factors. The results also suggest that fracture type, posterior medial cortex comminution, and reduction quality might be useful for evaluating postoperative neck shortening.

## Data Availability

All data analyzed during this study are included in this published article.
